# Cardiovascular risk factors and migraine without aura: A case-control study

**Published:** 2013

**Authors:** Samaneh Aalami Harandi, Mansoureh Togha, Azadeh Sadatnaseri, S. Hamed Hosseini, Soodeh Razeghi Jahromi

**Affiliations:** 1 Cardiology Resident, Department of Cardiology, School of Medicine, Isfahan University of Medical Sciences, Isfahan, Iran; 2 Professor, Department of Neurology AND Iranian Neurological Research Center, Sina Hospital, Tehran University of Medical Sciences, Tehran, Iran; 3 Department of Cardiology AND Sina Hospital, Tehran University of Medical Sciences, Tehran, Iran; 4 Department of Epidemiology and Biostatistics, School of Public Health AND Knowledge Utilization Research Center, Tehran University of Medical Sciences, Tehran, Iran; 5 Endocrine and Metabolic Research Center, Obesity group, Sina Hospital, Tehran University of Medical Sciences, Tehran, Iran

**Keywords:** Migraine, Hypertension, Aura, Lipid Profile

## Abstract

**Background:**

Migraine with aura (MA) has been identified as a risk factor for cardiovascular disease. Previous observation has also found higher prevalence of cardiovascular risk factors in migraineurs without aura (MO), but the results have been conflicting. The present study was conducted to assess the association between cardiovascular risk factors and migraine without aura among Iranians.

**Methods:**

In our study the prevalence of cardiovascular risk factors, including hypertension, hyperglycemia, dyslipidemia, obesity, cigarette smoking, and family history of early coronary artery disease, were studied in 347 migraineurs without aura and 267 non-migraineurs. The odds ratio (ORs) with 95% confidence interval (95% CI) was used to assess the strength of the association.

**Results:**

Patients with migraine without aura were at an increased risk of developing hypertension (OR_adj_ = 1.9; P = 0.029), but there was no difference in other cardiovascular risk profiles, including hyperglycemia, dyslipidemia, obesity, cigarette smoking, and family history of early coronary artery disease.

**Conclusion:**

Our study revealed that the prevalence of hypertension was higher in migraineurs without aura in comparison with non-migraineurs. Therefore, physicians are supposed to be more vigilant in examining these patients and take care not to prescribe medications that may provoke hypertension.

## Introduction

Headache and cardiovascular disease are highly prevalent, resulting in notable individual disability.^1, 2^ The association between migraine with aura (MA) and the risk of developing ischemic heart disease has been well established in previous studies.^3^ Recent evidence have also confirmed the link between MA and retinopathy, small vessel arteriolar intima thickening, endothelial injury, and impaired endothelial vasoreactivity.^2^ It seems that vascular comorbidities reported in migraineurs are limited to MA.^2^

Due to the shared pathology of migraine and cardiovascular disease, several studies have investigated the prevalence of cardiovascular risk factors in migraine sufferers.^2–5^ These studies have reported contradictory results. Some have shown an elevation in the Framingham risk score in both patients with migraine without aura (MO) and MA, whereas others have reported the condition only in MA patients. Moreover, few attempts have been made to examine the independent effect of each factor by adjusting for other risk factors. According to our knowledge, no study has yet assessed the relationship between MO and cardiovascular risk factors in the Iranian population. In this cross-sectional study, we assessed the prevalence of cardiovascular risk factors in MO patients.

## Materials and Methods

### Study population

All Patients aged between 18 and 55 years referred to an outpatient neurology clinic between September 2009 and February 2010 were recruited. This study was approved by the Ethics Committee of Tehran University of Medical Sciences. From among these patients 614 accepted to participate in the study and signed an informed consent according to the Helsinki treaty.

### Headache diagnoses

The diagnosis of headache was made by a neurologist expert in headache disorders in accordance with the International Headache Society (IHS) criteria for migraine without aura.^5^ These criteria were: (1) those experiencing headache attacks lasting for 4–72 h (< 4 h was accepted for those who reported visual disturbance before headache); or (2) those reporting headache with at least one of the following characteristics: pulsating quality, unilateral location or aggravation by physical activity; or (3) those experiencing at least one of the following symptoms during headache: nausea, photophobia, or phonophobia. Those who reported aura according to the IHS criteria were considered as MA and were excluded from the study. The control group comprised of patients, who did not suffer from any type of headache, with neurological problems, such as psychiatric disorders, epilepsy, and multiple sclerosis. Patients with any neurological disorder with confirmed vascular pathology, such as stroke or AVM, and those with a first degree relation within the case group were excluded.

Since all the cases and controls were recruited from the same medical center, they were believed to be socioeconomically matched. The age variable was tolerably matched (± 5 years). Considering the executive difficulties, female/male ratio was statistically different between the two groups; in multivariate analysis, however, it was eliminated as a confounder.

### Cardiovascular risk factors

Fasting blood sample was obtained to measure fasting blood sugar (FBS), total cholesterol, Low density lipoprotein (LDL), and high density lipoprotein (HDL). Hyperglycemia was defined as having FBS ≥ 126 mg/dL, or consuming oral hypoglycemic agents or insulin. The patient was diagnosed with dyslipidemia if he/she had serum levels of total cholesterol ≥ 240 mg/dL, LDL ≥ 160 mg/dL, HDL ≤ 40 mg/dL, or was taking lipid lowering drugs.

Blood pressure was measured on the upper arm with the participant in a sitting position after a 10-minute rest, using a Mercury Ricter ™ sphygmomanometer. If systolic (SBP) or diastolic blood pressure (DBP) were higher than 140 or 90 mmHg, respectively, the measurement was repeated after 30 minutes. Hypertension (HTN) was defined as having SBP ≥ 140 mmHg or DBP ≥ 90 mmHg at the time of examination, or personal history of consuming antihypertensive drugs.

Anthropometric measurements were made by weight and height conventional scales. Weight was measured using a digital scale (Omron, Omron Healthcare, USA) to the nearest 0.1 kg. Height was measured to the nearest 0.1 cm, using a tape meter. Obesity was described as having body mass index (BMI) (calculated by dividing weight in kilograms by height in square meters) ≥ 30 kg/m^2^.

A questionnaire including information on the patient's past medical history of cerebrovascular disease (CVD) and ischemic stroke, HTN, diabetes mellitus (DM), dyslipidemia and consumption of antihypertensive drugs, oral hypoglycemic agents, insulin and lipid lowering drugs was completed for all the participants. The history of cigarette smoking and family history of early CVD were also questioned. The history of cigarette smoking was considered positive if the individual had used even one cigarette during the last month.^7^ CVD history was considered positive if the participant reported a positive history of MI, coronary artery bypass graft surgery, or balloon or stent angioplasty.^7^ Participants were also asked about CVD history in their first-degree relatives before the age of 55 in males, or 65 in females.^7^

### Statistical analysis

For descriptive analysis of quantitative data, the Mean and Standard deviation were used. For qualitative data, frequency percentage was reported. To evaluate the association between Mo and risk factors, OR with 95% CI was used, and finally, to determine the real association of different variables, OR was adjusted by logistic regression model. We used multivariable logistic regression analysis to reduce the effect of confounding factors. The Statistical Package for Social Sciences (SPSS 15.0, SPSS Production Facility, Chicago, Illinois, USA) was used to analyze the data.

## Results

614 individuals were recruited. Male to female ratio was significantly lower in the case group (P = 0.02). Compared with the control group, migraineurs had higher age (P = 0.02), higher DBP (P = 0.01), lower FBS (P ≤ 0.001), higher total cholesterol (P = 0.006) and LDL (P = 0.006), and higher BMI (P = 0.02) (Table 1).

**Table 1:** Demographic characteristics of the studied population

Variables		Cases (n = 347)	Controls (n = 267)	P
Sex	Male	15.8%	23.2%	0.02
	Female	84.1%	76.7%	
Age(years)		36.1	34.2	0.02
SBP		120.3 ± 16.74	118 ± 18.52	0.12
DBP		78.3 ± 10.14	75.8 ± 11.38	0.01
FBS		95.7 ± 15.89	90.2 ± 16.95	≥ 0.001
Total Cholesterol		193.8 ± 35.95	182 ± 40.41	0.006
LDL		112.5 ± 33.27	107.7 ± 35.17	0.11
HDL		48.7 ± 10.58	47.2 ± 10.94	0.13
BMI		26.4 ± 5.12	25.5 ± 4.78	0.02

All continuous data is presented as mean ± SD (standard deviation)

HTN, hyperglycemia, dyslipidemia, obesity, and age were the probable confounders in the multivariate logistic regression analysis. The final model indicated HTN as the only variable related to MO (OR_adj_ = 1.9; P = 0.029). The odds ratio of the cardiovascular risk factors in the two studied groups is summarized in table 2.

**Table 2:** The crude odds ratio of the cardiovascular risk factors in the two groups

Risk factors	Cases	Controls	OR _crude_ (95% CI)	P

	No/valid numbers (%)	No/valid numbers (%)	
Ischemic stroke history	4/281 (1%)	0/245 (0%)	-	0.074
CVD history	1/283 (0.3%)	0/245 (0%)	-	0.525
Early familial CVD history	17/277 (6%)	12/252 (4%)	1.3 (0.6-2.7)	0.309
HTN	88/265 (33%)	38/245 (15%)	2.7 (1.7-4.16)	< 0.001
Hyperglycemia	17/208 (8%)	7/148 (4%)	1.8 (0.7-4.4)	0.144
Dyslipidemia	107/207 (51%)	93/164 (56%)	0.8 (0.5-1.2)	0.196
Obesity	59/275 (21%)	39/250 (15%)	1.4 (0.9-2.3)	0.054
Cigarette smoking	14/279 (5%)	22/254 (8%)	0.5 (0.2-1.1)	0.067

## Discussion

In line with previous observations, our study showed MO to be associated with a higher prevalence of cardiovascular risk factors in the Iranian population. After adjusting for other risk factors, only HTN was significantly more prevalent among MO sufferers.

One of the possible underlying mechanisms linking headache to HTN is the renin-angiotensin system. It should be mention that DD gene, elevated ACE activity, and higher rate of MO attacks were reported in those with angiotensin converting enzyme (ACE).^6^ Moreover, ACE inhibitors, such as captopril, and angiotensin II receptor blockers, such as lisinopril, have approved prophylactic effects in the treatment of migraine.^7^ Yetkin et al. also found lower brachial artery flow in patients with transformed headache.^8^ Another cross-sectional study reported a significant positive association between migraine and higher augmentation index (AI), a parameter of arterial stiffness.^7^ Similarly, Bigal et al. found a significant relationship between hypertension and MO.^3^ Ikeda et al., however, failed to report such an association.^9^

The results regarding the relationship between high blood glucose levels and headache were highly controversial. Some studies have found higher blood glucose levels in patients with headache, whereas others have reported diabetes to be less prevalent in headache sufferers.^2, 10^ Our study showed higher FBS levels in the MO group; however, after adjusting for other CVD risks, the difference was not significant. Possible explanations for this observation are that, on the one hand, insulin resistance may be involved in the pathogenesis of migraine, and on the other hand, individuals with high blood glucose levels are prone to diabetic neuropathy that could interfere with peripheral neurogenic mechanism necessary for triggering migraine.^10, 11^

According to previous studies, obesity, hyperlipidemia, and smoking can increase an individual's risk of experiencing headache.^2, 12^ Winsvold et al. showed BMI, serum total cholesterol levels, and smoking to have a significant direct relationship with MO.^2^ In the current study, only before adjusting for other CVD risk factors, the mean values of BMI and total cholesterol were higher in MO suffers. A possible explanation for the observed difference is the baseline differences noted in some of the CVD risk factors.

Considering the cross-sectional nature of the study, we cannot draw a conclusion as to a causal relationship between CVD risks and headache. Further prospective studies on larger populations are warranted.

## Conclusion

In conclusion, our study revealed that the prevalence of hypertension was higher in migraineurs without aura in comparison with non-migraineurs. Therefore, physicians must be more vigilant in examining these patients and prescribing medications that do not provoke hypertension.
